# Reiter’s syndrome as a manifestation of immune reconstitution inflammatory syndrome in an HIV infected individual

**DOI:** 10.1186/1471-2334-14-S3-E17

**Published:** 2014-05-27

**Authors:** J Shankari, V Sudha, K Manoharan

**Affiliations:** 1 Department of Dermatology, Institute of Venereology, Madras Medical College, Chennai, India

## Introduction

Reiter's syndrome is a triad of arthritis, urethritis, and conjunctivitis. The prevalence of Reiter's syndrome in HIV infected patients varies between 1.7% and 11.2%. This syndrome can also occur as a part of immune reconstitution inflammatory syndrome (IRIS).

## Case

A 50 year old male, known HIV patient on ART for past 4 months, presented with complaints of scaly raised lesions over palms, soles and genitalia with history of burning micturition and swelling of right knee for over one month. On examination patient was thin built, anaemic. Right knee joint effusion with tenderness was present. Palms and soles revealed keratoderma blenorrhagicum. Nails were yellowish and dystrophic. Oral cavity showed candidiasis and oral hairy leukoplakia. Genital examination showed circinate balanitis. Clinical diagnosis of Reiter’s syndrome was made. Haemoglobin was low, ESR and CRP were elevated. CD4 counts were raised when compared to previous values. RNA load showed 2 log decreases. Urine culture showed no growth. Chest x ray had features of tuberculosis. Symptomatic improvement was seen after treatment with ofloxacin, isotretinoin and indomethacin.

## Discussion

In HIV infected patients, Reiter’s syndrome may follow a severe clinical course after immune reconstitution associated with ART. However, because of possible higher antigen dependence, clinical outcome may be more favourable if a specific infectious agent can be identified and treated with antibiotics.

## Conclusion

We are presenting this case to highlight the occurrence of Reiter’s syndrome as a rare manifestation of IRIS in an HIV infected individual.

